# Zero-Shot Human Activity Recognition Using Non-Visual Sensors

**DOI:** 10.3390/s20030825

**Published:** 2020-02-04

**Authors:** Fadi Al Machot, Mohammed R. Elkobaisi, Kyandoghere Kyamakya

**Affiliations:** 1 Research Center Borstel—Leibniz Lung Center, 23845 Borstel, Germany; 2 Institute for Applied Informatics, Application Engineering, Alpen-Adria University, 9020 Klagenfurt, Austria; M3mohammed@edu.aau.at; 3 Institute for Smart Systems Technologies, Alpen-Adria University, 9020 Klagenfurt, Austria; kyandoghere.kyamakya@aau.at

**Keywords:** activity recognition, sensor data, zero-shot learning, non-visual sensors

## Abstract

Due to significant advances in sensor technology, studies towards activity recognition have gained interest and maturity in the last few years. Existing machine learning algorithms have demonstrated promising results by classifying activities whose instances have been already seen during training. Activity recognition methods based on real-life settings should cover a growing number of activities in various domains, whereby a significant part of instances will not be present in the training data set. However, to cover all possible activities in advance is a complex and expensive task. Concretely, we need a method that can extend the learning model to detect unseen activities without prior knowledge regarding sensor readings about those previously unseen activities. In this paper, we introduce an approach to leverage sensor data in discovering new unseen activities which were not present in the training set. We show that sensor readings can lead to promising results for zero-shot learning, whereby the necessary knowledge can be transferred from seen to unseen activities by using semantic similarity. The evaluation conducted on two data sets extracted from the well-known CASAS datasets show that the proposed zero-shot learning approach achieves a high performance in recognizing unseen (i.e., not present in the training dataset) new activities.

## 1. Introduction

Recognizing daily activities in smart environment requires various devices to collect data from sensors like cameras, video recordings, static images, and other activity recording/detection sensors. Activity recognition-based sensors (non-audio/visual sensors) have a series of specific advantages compared with other devices due to the characteristics of their sensor equipment such as low-cost, less-intrusive, privacy, and security preserving nature [1,2]. These advantages make these sensors more acceptable by users, and thus widely used in activity recognition concepts and related machine learning algorithms [3,4,5,6,7,8,9]. However, traditional methods mostly use supervised machine learning to recognize human activity that require both training data and corresponding labels for each activity involved. Training data recording and activity-labeling are often time-consuming and costly to obtain, as it demands a huge effort from test subjects, annotators, and domain experts. Therefore, it has been reported that a fully supervised learning method, where labeled instances from different contexts are provided to the system, may not be possible for many applications [10,11].

Furthermore, the existing approaches to activity recognition cannot recognize a new activity which is not presented in its training set. According to the activity lexicon in the ATUS survey [12], there are at least 1104 different activities that people perform in their everyday life. Considering the differences between individuals, cultures, and situations that were not covered by the study, the actual number of the activities is likely to be more. However, the primary drawback of the existing activity recognition-based sensors is that they prevent systems from recognizing any previously invisible activities. On the other hand, sensor data contain rich semantic relationships that can be appropriately investigated to the estimate and or detect novel (i.e., not previously seen in the training set) activities. Considering the lastly described existing limitations, there are key research questions we aim to answer in this paper:

**Q1.** How do we exploit the advantages of involving specific sensors types in the activity recognition process (e.g. low cost, less intrusive, and privacy preserving)? **Q2.** How do we embed sensor readings data to predict or recognize previously unseen new activities? **Q3.** How do we recognize high-level activity labels when there is no related data in the training model? **Q4.** Is it possible to apply zero-shot activity recognition using only a small data sample amount in the training set?

In this paper, we present a technique to recognize activity events when there is no related training data for a target activity by utilizing the previous knowledge on the existing activities. Herein, we have developed the current approach to tackle the above-mentioned research questions. We involve heterogeneous knowledge from sensor data and semantic word space, which is extracted from low-level sensor data and uses some external machine learning method.

In particular, we refer here to the zero-shot learning, where the classes covered by training and testing samples are disjoint [13,14].

Zero-shot learning has recently demonstrated promising performance results according to the latest computer vision literature [15]. This type of learning enables the prediction (or detection) of newly observed activity type or calls by using semantic similarity between the activity and other embedded words in the semantic space. It uses the so-called “word2vec” tool [16] for modeling latent semantic text by taking each activity-label as an input and then producing a corresponding high dimensional vector space [17].

For example, assume that we have training data for two activities “CookingLunch” and “WashingDishes”. If we want to detect a new activity “EatingLunch” rather than hiring subjects to collect and annotate a new activity, our approach employs semantic similarity to predict a new activity by reusing the model that has already been trained on two known activities. Still, there are several challenges that must be overcome to apply zero-shot learning in activity recognition for the following reasons. (1) Most of the previous works on zero-shot learning focused on images and videos, which are totally different from other forms of sensor data; (2) sensor data are generally noisy, and the noise may change the relationship between the features and the desired output; (3) it is not clear which features within the sensor data are useful to recognize activities; and (4) to train a normal model, one generally needs a large amount of sensor data, but in our case, we consider situations where one has only a small amount of sensor data for training. To address the above formulated research questions Q1, Q2, and Q3, we have designed a representation for human activity recognition through correlating high-level activity-labels by embedding semantic similarity. The description of semantic similarities is based on low-level features captured from event occurrences in the sensor data. For research question Q4, to improve the recognition accuracy, we compared the output using various scenarios to reach the best recognition performance. We summarize our core contributions in this work as follows.

- Designing a method to recognize human activity from non-visual sensor readings instead of traditional methods, which depends on images or video observations.
- Combining both the semantic similarity and zero-shot algorithms for robustly recognizing previously unseen human activities.
- Implementing our approach by using different training and testing samples to enhance and validate the good/high recognition accuracy of previously unseen activities.
- Evaluating the system through the use of two well-known public activity recognition datasets.

Furthermore, the suggested system may also help the machine to gain a deeper understanding of activity patterns such as a person’s long-term habits. Additionally, it can be used to motivate a person to add new healthy activities into his/her normal routine to improve the quality of his life. Unlike recognition concepts using only pure low-level features of sensor data, semantic similarity makes the activity recognition task more reliable, especially when the same activity may look different due to the variety of activities performed. Additionally, this method may also be useful for those scenarios where the training model has been learned for recognizing activities in one smart house and then be used/utilized later in another house. The rest of this paper is structured as follows. In Section 2, we discuss and compare the related works. In Section 3, we give a detailed description of our novel method. In Section 4, we present the datasets used in the various experiments. Then, Section 5 presents the evaluation methodology used and then discusses the results obtained. Finally, in Section 6, we present a comprehensive summary of the quintessence of the paper contribution and the core results obtained. The paper ends with a series of concluding remarks along with a comprehensive outlook w.r.t. future subsequent works in Section 7.

## 2. Related Works

In this section, we briefly review the prior works or relevance and do group them into three main directions. To avoid the redundancy, we briefly reviewed the state-of-the-art, considering the diversity of underlined method, activity, input source, and the highest performance, as follows.

### 2.1. Activity Recognition-Based Supervised Learning

Regarding human activity recognition approaches, most of the related published studies address such a recognition using supervised learning [18,19,20,21,22] or semisupervised learning [23,24]. Transfer learning has also been investigated, whereby the instances or models for activities in one domain can be transferred to improve the recognition accuracy in another domain for the purpose of reducing the need for training data [25,26,27]. Although many promising results have been achieved, a widely acknowledged problem is that labeling all the activities is often very expensive, as it takes a lot of effort for the test subjects, human annotators, domain experts, and it does nevertheless remain error-prone [28,29]. However, providing accurate and opportune information is one of the most important tasks in identifying human activity. A lot of studies are based on supervised learning for recognizing human activity, some of which are summarized in Table 1. In this table, one compares the classification techniques, the different activities, the input sources, the respective observed performance, and finally the best performance that has been achieved using a particular classifier.

Furthermore, activity recognition has been widely reported in many fields using sensor modalities, including ambient sensors [35], wearable sensors [36], smart phones [34], and smart watches [37]. Those sensors contribute to developing a wide range of application domains such as sport [38], human–computer interaction [39], surveillance [40], video streaming [41], healthcare system [42], and computer vision area [43]. Due to the properties of noninvasive sensors, some studies discussed how to monitor human activities using this type of sensors (i.e., non-visual sensors) because they are both easy to install and privacy preserving [44,45].

Regarding supervised learning, it should be mentioned that Deep Learning has been applied for human activity recognition. Table 2 overviews previous works for recognizing activity using different sensors.

### 2.2. Activity Recognition-Based on Zero-Shot Learning

Zero-shot learning is an extended form of supervised learning to solve classification problems where there are not enough (i.e., only few) training instances are available for all classes. It depends on reusing the semantic knowledge between seen and unseen classes [50]. The notion of zero-shot learning was firstly presented in the field of computer vision [51,52,53]. The goal was to teach a classifier to predict novel classes that were omitted from the training set. After that, a lot of works have emerged [54,55,56,57]. To predict human activity, the major applications were related to visual attributes acquired from image or video sources. Table 3 presents prior studies on zero-shot human activity-recognition that depict different accuracy levels. The prediction of the next activity was also investigated in the work [58] to provide better assistance for elderly people. Unlike in the aforementioned studies, the focus in this last cited work was on predicating a next action based on the behavior history of a person.

Despite the fact that a significant progress has been made in zero-shot based activity recognition in the last years, it is unfair to compare their performance with that of supervised learning because the zero-shot concepts used to recognize activities have never been never seen before.

Today, there is a tendency towards using a noninvasive and non-visual activity sensing to collect information and infer activity without disturbing the person, as nobody wants to be constantly monitored and recorded by cameras. Additionally, they (i.e., non-visual sensors) are more flexible computing resources. It is indeed difficult to attach video recorders to a target subject to collect body information during daily activities. Besides, video or image processing methods are comparatively more expensive and time-consuming.

However, as we mentioned earlier, the limitations of the previous studies compared to our work is that the existing activity recognition-based supervised learning methods still cannot recognize a previously unseen new activity if there are no training samples of that activity in the dataset. Besides, the existing studies on activity recognition-based zero-shot learning focus mostly on images and videos as inputs, which is quite different from recognition involving non-visual sensor data. Due to the availability of huge samples sizes and rich features in the concepts that use images or videos, it is much easier for them to identify activities when compared to a noninvasive and non-visual sensor that relies thereby also only on very few samples.

## 3. Proposed Framework

**Figure 1:**
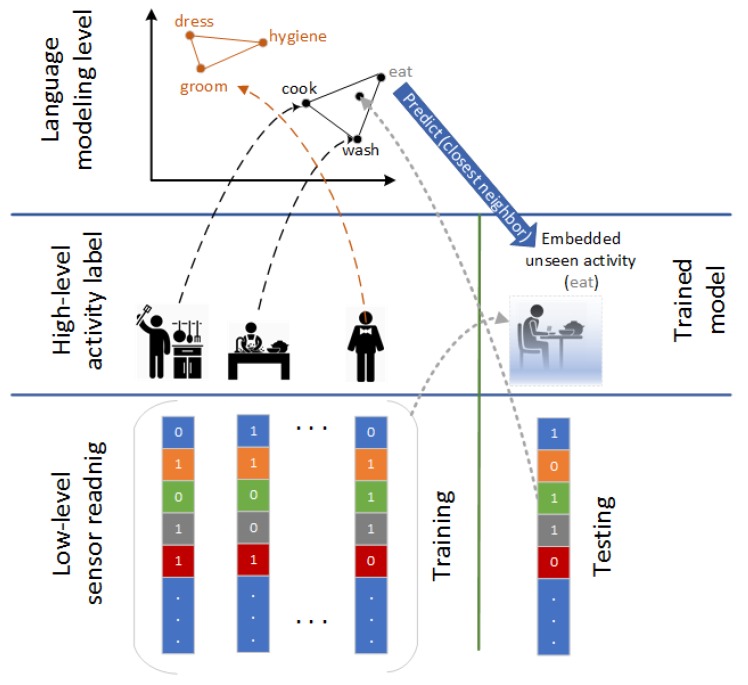
Main idea of proposed method.

In our framework, the features of each activity are extracted from corresponding sensor readings using fixed-length trained dataset. Due to the dissimilarity between trained and tested activities, a mapping from a different space is required to infer high-level activity labels. Predicting a new label for hidden activities is supported by a language modeling level that presents the nearest embedded words matching the target activity in the shared semantic space (see Figure 1). We have initialized the word embedding with a pre-trained embedding Google-News dataset. We specify word vectors of 300-dimensions for each trained activity [62].

As we have described before, the training and testing instances in zero-shot learning have different lengths $N_{train}\neq N_{test}$. Furthermore, the intersection between “already seen” and “previously unseen” activities is empty $N_{train}\cap N_{test}=\phi$.

### 3.1. Problem Definition

Assume a labeled training set of *N* samples is given as $D_{tr}=(X_{tr},Y_{tr},T_{tr}),tr=1,\ldots ,N$, with an associated class label set $T_{tr}$, where $x_{tr}$∈$X_{tr}$ is the tr-th training activities (sensor readings), $Y_{tr}$∈$R^{L\times 1}$ is its corresponding L-dimensional semantic representation vector, and $t_{tr}$∈$T_{tr}$ is the the training class label. We have $T_{tr}\cap T_{ts}=\phi$, i.e., the training (seen) classes and test (unseen) classes are disjoint. Note that each class label is associated with a predefined semantic space representation $Y_{tr}$ and $Y_{ts}$ (e.g., attribute vector), referred to as semantic class prototypes. Given a new test activity $x_{ts}\in X_{ts}$ and $Y_{ts}$ which is the corresponding L-dimensional semantic representation vector, the goal of zero-shot learning is to predict a class label $t_{ts}\in T_{ts}$.

### 3.2. Preprocessing Procedure

Sensor data are represented as a sequence of events, and every change in a sensor state (i.e., value) generates an event. All sensor readings/events (SE) produce binary values: ON/OFF motion sensors, OPEN/CLOSE door sensor, and/or numeric values for environmental sensors (e.g., temperature, humidity, light, etc.). These events are used to extract/infer complex activities. As a result, we have a matrix $R^{m\times n}$, where *m* is the number of activities and *n* is the dimensionality of the data. The construction (or generation) of the data relies on the fact that when the sensor (s) turns “ON”, its value is set to 1, and then to 0 when its value is “OFF”. In this case, we count how many times ONs occurred in a specific activity. A preprocessing of raw data consists of multiple-steps as shown in Algorithm 1.
**Algorithm 1:** Preprocessing procedure. 
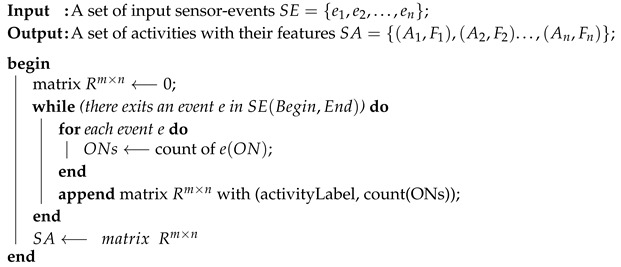


### 3.3. Approach

As we have already previously explained it, zero-shot learning is an extension of the supervised learning to overcome a well-known problem in machine learning when too few labeled examples are available for all classes.

We collect sensor readings for training classes and thereby, we can get them for all available activity samples. However, we do not have any sensor readings samples for zero-shot classes, and we do not even know how they look like. Additionally, as zero-shot activities are not involved in the training phase, a different and appropriate data representation for the zero-shot and training activity labels is required, which will function as a bridge between training and zero-shot classes. This data representation should be generated from all data samples by ignoring that they belong either to training classes or to zero-shot classes.

**Figure 2:**
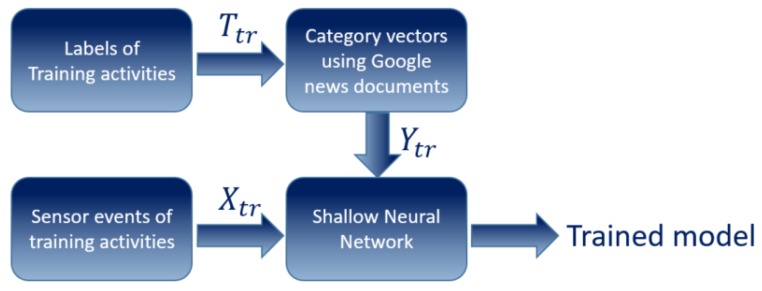
Training phase, where $T_{tr}$ are the training class category labels, $X_{tr}$ are the sensor readings of training activities, and $Y_{tr}$ are the corresponding L-dimensional semantic representation vectors of the training labels.

Concerning activity labels embedding, we use Google Word2Vec representation trained on Google News documents (https://code.google.com/archive/p/word2vec/). We consider a Wor2Vec of 300 dimensions for each of the training classes we have specified. Therefore, the algorithm is structured as follows [52] in the training phase (see Figure 2).

1. Given some known training class category labels $T_{tr}$, the sensor readings of training activities $X_{tr}$ and the corresponding L-dimensional semantic representation vectors of the training labels $Y_{tr}$.
2. Learn activities using a shallow neural network model $F(X_{tr}$,$Y_{tr})$.

**Figure 3:**
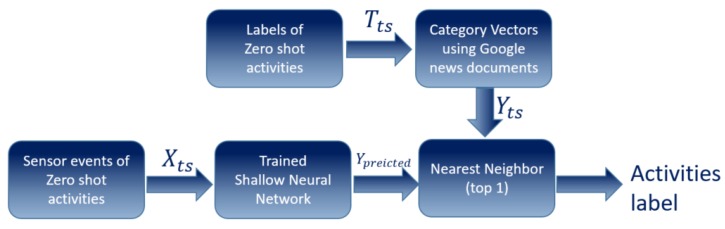
Test phase, where $T_{ts}$ are the zero-shot class labels, $X_{ts}$ are the sensor readings of zero-shot activities, and $Y_{ts}$ is its corresponding L-dimensional semantic representation vector of the zero-shot class labels.

In the test phase, which means the recognition phase (see Figure 3):

1. Given online sensor readings of a new unseen activity $X_{ts}$ which has not been used in training
2. Map test data $X_{ts}$ to category vector space $Y_{ts}$
3. Apply nearest neighbor matching of $Y_{ts}$ vs $Y_{predicted}=F\left(X_{ts}\right)$

### 3.4. Classification Model

**Figure 4:**
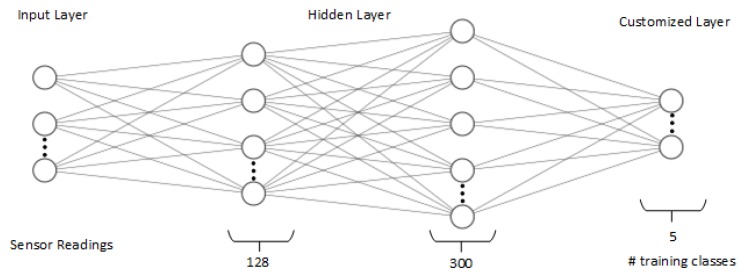
The proposed shallow neural network model.

The aim of this model is to map inputs (sensor readings) to corresponding outputs (Word2Vecs). To perform the classification task, we use a shallow neural network neural model [63,64]. The shallow neural network model consists of four layers, input layer, two hidden layers, and an output layer. First, the sensor readings of training activities (input layer) are fed into the two hidden layers in the neural model which consist of 128 neurons and 300 neurons, respectively, supported by an Exponential Linear Units (SELU) activation function, then the final layer is the output layer which consists of a softmax activation function and its size is related to the number of training activity classes. Adam optimizer [65] has been used which is an adaptive learning rate optimization algorithm that has been designed specifically for training deep neural networks. The parameters are selected by using grid search from scikit-learn library (see Figure 4).

The proposed shallow neural network model has been trained on both datasets (see Section 4). Additionally, we should mention that batch normalization is taken into consideration during training and the last layer has been customized.

The goal of the customized layer is that the weights should be initialized using Word2Vecs of the training activities and the layer should not be trainable. Namely, it should be a simple matrix multiplication placed at the end of the network.

### 3.5. Evaluation Metrics

To evaluate the overall performance of the classifiers, we consider several performance metrics. In particular, we use precision, recall, f-measure, and accuracy, as in [66].

The Equations (2)–(4) show mathematical expressions of the metrics precision, recall, accuracy and f-measure respectively, where TP, TN, FP, and FN refer respectively to “True-Positives”, “True-Negatives”, “False-Positives”, and “False-Negatives”, respectively.

(1) $$Accuracy=\frac{\mathrm{TP}\;+\;\mathrm{TN}}{\mathrm{TP}\;+\;\mathrm{TN}\;+\;\mathrm{FP}\;+\;\mathrm{FN}}$$

(2) $$Precision=\frac{\mathrm{TP}}{\mathrm{TP}+\mathrm{FP}}$$

(3) $$Recall=\frac{\mathrm{TP}}{\mathrm{TP}+\mathrm{FN}}$$

(4) $$F1=\frac{2\cdot precision\cdot recall}{precision+recall}$$

## 4. Datasets Description

**Figure 5:**
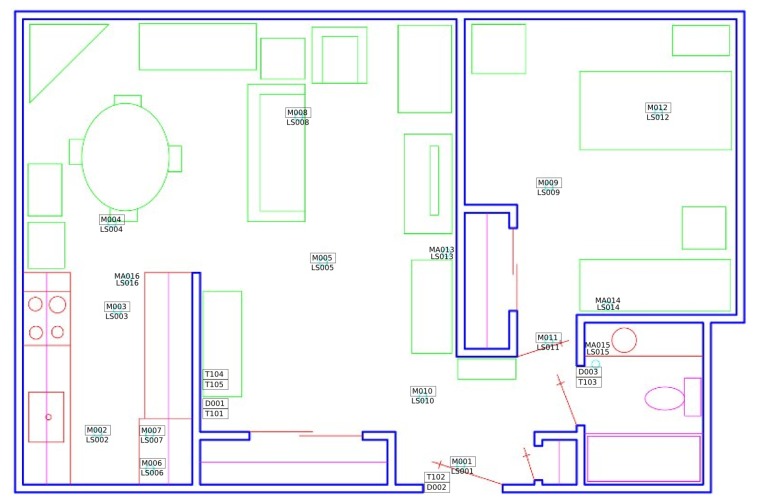
Layout of HH101 apartment. The position of each sensor is specified with the corresponding motion (M), light (LS), door (D), temperature (T), or sensor number.

We selected two datasets (HH101, HH125) obtained in the CASAS (http://casas.wsu.edu) smarthomes that reflect daily activities in the real-world using sensor streams. In the HH101 dataset, there are 30 distinct activities belong to one subject and 76 sensor types. HH125 dataset includes 34 activities performed by single-resident apartment and 27 different sensors. However, both datasets contain sensor readings indicating the beginning and ending of an activity. In the evaluation, we chose some appropriate activities to demonstrate our concept. Table 4 compares the total number of each activity that is used in our experiment for both HH101 and HH125 smart-homes. Home layout and sensor placement of each dataset is different. Each house is equipped with a combination of different types of sensors deployed in different locations (e.g., battery levels, motion, temperature, door, and light sensors). Figure 5 shows a sample layout and sensor placement for HH101 smarthome.

The raw data from various sensor readings are filtered and preprocessed to extract low-level features. This process is based on sensor status, either ON/OFF or a numeric value. The resulted low-level features transform into embedded vector to examine contextual word similarity. The nearest similar word is computed to classify activity label despite the fact that there is no training data for that activity. The size of vector space can be specified to a particular dimension, in our case, 300-dimensional vectors.

## 5. Results

We evaluate our method with datasets collected from households using simple data sensors. In this section, we investigate whether an activity that has never been seen in the training set is predicted correctly and how the system’s performance changes when various scenarios are observed. In order to evaluate the activity recognition approach, we selected two scenarios using two well-known benchmark datasets. Moreover, four performance measures are considered: Accuracy, Precision, Recall and F-Measure. They are calculated to give a full evaluation of the performance of our proposed system. Table 5 shows two scenarios: the first scenario “scenario 1” uses activities such as “bathe, cook, wash dinner dishes, watch TV, and read” for training, and activities such as “sleep, toilet, and relax” for zero-shot. The table also shows the second scenario “scenario 2”, which uses activities such as “cook breakfast, wash dishes, phone, dress, and eat dinner” for training, and activities such as “ cook breakfast, personal hygiene, and eat lunch” for zero-shot, respectively.

In the previous scenarios, note that there are semantic relations between activities. For example, in scenario 1:

- bathe may relate to sleep and dinner
- toilet may relate to wash
- relax may relate to read, watch, and bathe

### 5.1. Scenario 1

Table 6 shows the confusion matrix for scenario 1 using the HH101 and HH125 dataset. However, for the HH101 dataset, there are false positive cases between some toilet and sleep activities. Table 7 shows the performance metrics for scenario 1 using the HH101 dataset and the HH125. It can be seen that the proposed approach achieved the best accuracy for relax activity and toilet for HH101 and HH125 datasets, respectively. Furthermore, for both datasets HH101 and HH125, some relax activities are recognized as sleep.

### 5.2. Scenario 2

Table 8 shows the confusion matrix for scenario 2 using the HH125 and HH101 datasets. However, there are some false-positive cases between personal hygiene activities and cook lunch, and between personal hygiene and eat lunch for HH101 and HH125, respectively. Table 9 shows the performance metrics for scenario 2 using the HH101 and the HH125 datasets. It can be seen that the proposed approach achieved the best accuracy for eat lunch and cook lunch for HH101 and HH125 datasets, respectively.

Regarding the false-positives related misclassification, this is due mostly to (a) activities that share the same sensors (e.g., relax and sleep or toilet and sleep); (b) activities that have very close word vectors in the embedding space; and (c) sensors that are allocated close to each other although related to the zero-shot activities.

## 6. Discussion

We have used sensor-semantic embedding for zero-shot and addressed the problems associated with the framework that are specific for zero-shot learning. Based on the research question (Q1), our method has shown success in utilizing the characteristics of simple sensor readings, by embedding (Q2) the semantic information of the sensors and the activities. The classification model (Q3) is learned with a data set and used to recognize the activity that has never appeared in a testing set, which has different label and sensor readings. Experiments on real-world small data learning (Q4) show the effectiveness of the proposed zero-shot activity recognition.

Despite the success of the standard zero-shot learning, there are some challenges that limit its performance.

1. The majority of zero-shot models ignore that the semantic space is highly subjective, as they are created by a human or automatically extracted. It may not be complete or discriminatory enough to classify different classes because of the scarcity of similar seen classes which describe the unseen.
2. There is a semantic gap between existing semantic space and the ideal space, because the model trained on huge possible words, for example GoogleNews or Wikipedia, may contain unrelated texts. This may raise concerns about the validity of the results.
3. Zero-shot suffers from well-known: hubness [67,68,69] and bias problems [53,70,71]. Due to these problems, the models sometimes perform poorly towards unseen classes.
4. In a real-world setting, an appropriate sensor data segmentation concept that can define a robust windowing approach for human activities’ recognition is still a challenging issue. Hereby, when dealing with the inputs (which are the sensor data) in real-life (i.e., online), a possible solution proposed by the approach in [72], which follows the so-called best-fitting sensors strategy. The proposed approach consists of two phases: (a) an offline phase where the best-fitting sensors are selected for each activity by using the related respective information gain, and (b) an online phase (or real-life phase), which defines a windowing algorithm that segments sensor readings to be given as input to the support vector machine classifier. Basically, a window is so selected that all best-fitting sensors are activated for any given activity.

However, in this paper, our objective is not to overcome the above-mentioned challenges. Instead, we exploit the benefits of this important algorithm in predicting unseen activities.

**Figure 6:**
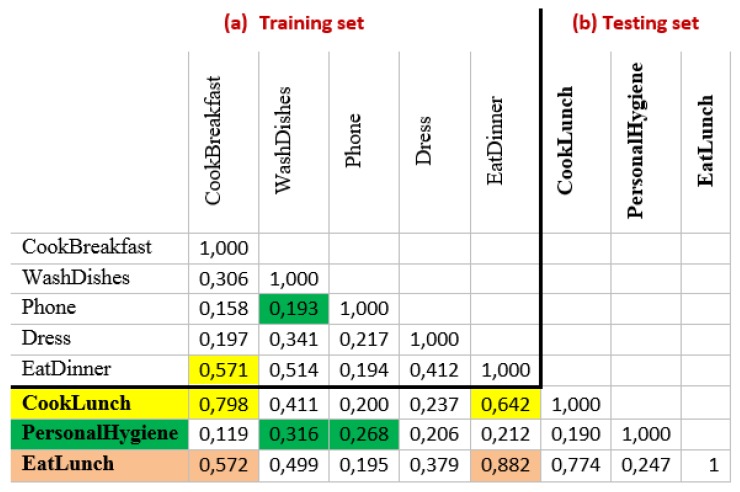
Correlation between seen and unseen activities in scenario (one). (**a**) Training set; (**b**) Testing set.

It is a complex task to observe model behavior on unseen activities by training the model on seen activities, as it is highly probable that it receives misclassification. In our empirical evaluations, we identify several pertinent issues that underpin zero-shot in recognition task. Moreover, we have computed the correlation between seen and unseen activities, which resulted in zero-shot recognition (see Figure 6).

We observed that

1. The correlation between seen classes “that infer unseen” must be less than the correlation between unseen and seen ones (e.g., corr(CookBreakfast, EatDinner) < corr(CookLunch, CookBreakfast), and < corr(CookLunch, EatDinner)).
2. To obtain a better result, the correlation in the seen training set must be spaced. For example, when phone activity is discarded in training, it will lead to poorly unseen result, as the semantic space is small.
3. It is difficult to anticipate to which seen activity the unseen belongs if the distance between “seen” instances is very close. E.g., CookLunch+EatLunch to infer WashLunch.
4. Generally, in both training and testing sets, the semantic relationship between samples should be small. In other words, the distance between samples, $Dis\left(x,y\right)=\frac{x\cdot y}{\left||x||\cdot ||y|\right|}$ must not be small.
5. Since the semantic space contains a huge number of similar words, the recognition task is more susceptible to predict an incorrect label (e.g., wrongly predicting a “WashHands” as “WashDishes”, where both are semantically similar, because they belong to washing activities).
6. Exploiting less labeled data in real life to recognize more activities involves several challenges. As a potential solution, a study [73] proposes a practical way to predict data labels outside the laboratory.

We should mention that zero-shot activities should also be classified correctly when there is a large number of unseen categories to choose from. To evaluate such a setting with many possible but incorrect unseen classes, we may create a set of distractor words. We compare two scenarios: in the first, we add random nouns to the semantic space. In the second, a much harder setting, we add the k-nearest neighbors of a word vector. As a result, the accuracy should not change much when random distractor nouns are added. Such an experiment can show that the semantic space is spanned well and our zero-shot learning model is quite robust.

However, regarding the issue of comparing the proposed approach, which is based on non-visual sensors with other zero-shot approaches, which are based on visual data (such as videos and images as shown in Table 3), we can state the following. (a) Our high/better performance is due to the fact that the input dimension of our sensors’ readings is much smaller than that of sensor visual data, and (b) the complexity of visual data is much higher than that of non-visual sensors data especially w.r.t., for example, noise, enhancement, and restoration. Generally, in [74], several researchers have already addressed the accuracy of various zero-shot learning approaches using visual datasets, e.g., Animal with Attributes(AwA) [75], aPascal and aYahoo (aPY) [76], Caltech-UCSD Birds-200-2011 (CUB) [77], and SUN [78]. Those authors have mentioned that the Joint Latent Similarity Embedding (JLSE) approach showed a promising accuracy, e.g., 80.46%, 50.35%, 42.11%, and 83.83% for AwA, aPY, CUB, and SUN, respectively. However, another approach proposed in [74], which is based on formulating a softmax-based compatibility function and an improved optimization technique showed better accuracy, e.g., 84.50%, 42.40%, 48.10%, and 85.50% for AwA, aPY, CUB, and SUN, respectively.

## 7. Conclusions

Due to the cost of obtaining human generated activity data and similarities between existing activities, it can be more efficient to reuse information from existing activity recognition models instead of collecting more data to train a new model from scratch. In this paper, we have presented a method for integrating low-level sensor data with semantic similarity of word vectors to infer unseen activities depending on seen ones. We applied zero-shot learning to estimate occurrences of unseen activities. Furthermore, we have presented several challenges that must be taken into account when selecting training and testing samples using the suggested zero-shot learning. Experimental results show that our approach has achieved a promising accuracy for unseen new activities’ recognition. As a future work, to confirm our hypothesis, we have to train our model with various combinations of activities. We also plan to integrate different machine learning algorithms to improve system performance. Moreover, we will extend our evaluation to train activity samples in one smarthome environment and predict unseen activity in a different environment.

## Figures and Tables

**Table 1:** Overview of activity recognition based on classical machine learning approaches. k-NN: k-Nearest Neighbor; SVM: Support Vector Machine; RF: Random Forest; MLP: Multi-Layer Perceptron; GMM: Gaussian mixture model; KF: Kalman Filter.

Paper	Approach	Method	Activity	Input Source	Performance
[30]	Comparison study to classify human activities	SVM, MLP, RF, Naive Bayes	Sleeping, eating, walking, falling, talking on the phone	Image	86%
[31]	Hybrid deep learning for activity and action recognition	GMM, KF, Gated Recurrent Unit	Walking, jogging, running, boxing, hand-waving, hand-clapping	Video	96.3%
[32]	Infer high-level rules for noninvasive ambient that help to anticipate abnormal activities	RF	Abnormal activities: agitation, alteration, screams, verbal aggression, physical aggression and inappropriate behavior	Ambient sensors	98.0%
[33]	Active Learning to recognize human activity using Smartwatch	RF, Extra Trees, Naive Bayes, Logistic Regression, SVM	Running, walking, standing, sitting, lying down	Smartwatch	93.3%
[34]	Recognizing human activity using smartphone sensors	Quadratic, k-NN, ANN, SVM	Walking upstairs, downstairs	Smartphone	84.4%

**Table 2:** Overview of activity recognition based on Deep Learning. SVM: Support Vector Machine; RBM: Restricted Boltzmann Machine; k-NN: k-Nearest Neighbor.

Paper	Approach	Method	Activity	Input Source	Performance
[46]	Mapping of activity recognition to image classification task	AlexNet, CaffeRef, k-NN, SVM, BoF	Communicating, sleeping, staying, work at computer, reading, writing, studying, eating, drinking	Image	90.78%
[47]	Recognizing activity using triaxial accelerometers and deep learning	RBM	Jogging, walking, upstairs, downstairs, sitting, standing	On-body sensors	98.23%
[48]	Deep CNN for recognizing activity using smartphone sensors	SVM, ConvNet, FFT	Walking, W. Upstairs, W. Downstairs, Sitting, Standing, Laying	Smartphone	95.75%
[49]	Smartwatches and deep learning to recognize human activity	RBM	(Gesture-based activity recognition), (Physical activities: Walking upstairs, downstairs), and (Indoor/Outdoor routine activities)	Ambient sensors, Smartwatch	72.1%

**Table 3:** Overview of activity recognition-based zero-shot learning. BGRU: Bidirectional Gated Recurrent Unit; GloVe: Global Vectors; ConSE: Convex Combination of Semantic Embeddings.

Paper	Approach	Method	Activity	Input Source	Performance
[59]	Zero-Shot activity recognition using visual and linguistic attributes	BGRU, GloVe	Drink, uncork, drool, lick	Image	42.17 %
[60]	Zero-shot activity-recognition based on a structured knowledge graph	Two-stream GCN method, self-attention mechanism	Biking, Skiing	Video	59.9%
[55]	Identify the hierarchical and sequential nature of activity data	Graphical Model of Semantic Attribute Sequences	ArmUp, ArmDown, ArmFwd, ArmBack, ArmSide, ArmCurl, SquatStand	Sequence of signal features	70–75%
[61]	Probabilistic framework for zero-shot action recognition	Inductive setting for standard zero-shot	(101+51+16) classes from different datasets	Video	57.88 ± 14.1%
[57]	Enable fair use of external data for zero-shot action recognition	ConSE	(51) and (400) classes from two datasets	Video	25.67 ± 3.5%

**Table 4:** Count of activities in HH101 and HH125 smarthomes.

Activity	HH101	HH125
Bathe	59	25
Cook	13	19
Cook Breakfast	79	78
Cook Lunch	18	65
Dress	139	212
Eat Dinner	22	10
Eat Lunch	14	8
Personal Hygiene	154	219
Phone	37	57
Read	53	19
Relax	92	9
Sleep	284	178
Toilet	369	287
Wash Dinner Dishes	18	100
Wash Dishes	31	154
Watch TV	333	218

**Table 5:** Training vs. zero-shot classes.

Dataset	Training	Zero-Shot
	Bathe	Sleep
	Cook	Toilet
Scenario 1s	Wash Dinner Dishes	Relax
	Watch TV	
	Read	
	Cook Breakfast	Cook Lunch
	Wash Dishes	Personal Hygiene
Scenario 2	Phone	Eat Lunch
	Dress	
	Eat Dinner	

**Table 6:** Confusion matrix for zero-shot activity recognition—Scenario 1.

Dataset	Activity	Relax	Sleep	Toilet
	**Relax**	84	0	0
HH101	**Sleep**	7	79	0
	**Toilet**	0	97	352
	**Relax**	1	0	0
HH125	**Sleep**	7	95	0
	**Toilet**	0	97	253

**Table 7:** Performance metrics for zero-shot activity recognition—Scenario 1.

Dataset	Class	N (Classified)	N (Truth)	Accuracy	Precision	Recall	F-Measure
	**Relax**	91	84	98.87	1	0.92	0.96
HH101	**Sleep**	176	86	83.2	0.92	0.45	0.6
	**Toilet**	352	449	84.33	0.78	1.0	0.88
	**Relax**	8	1	98.03	1.0	0.13	0.22
HH125	**Sleep**	95	102	98.03	.93	1.0	0.96
	**Toilet**	253	253	100	1.0	1.0	1.0

**Table 8:** Confusion matrix for zero-shot activity recognition—Scenario 2.

Dataset	Activity	Cook Lunch	Eat Lunch	Personal Hygiene
	**Cook Lunch**	13	0	0
HH101	**Eat Lunch**	0	14	0
	**Personal Hygiene**	4	0	154
	**Cook Lunch**	64	0	0
HH125	**Eat Lunch**	0	1	0
	**Personal Hygiene**	0	6	219

**Table 9:** Performance metrics for zero-shot activity recognition—Scenario 2.

Dataset	Class	N (Classified)	N (Truth)	Accuracy	Precision	Recall	F-Measure
	**Cook Lunch**	17	13	97.84	1.0	0.76	0.87
HH101	**Eat Lunch**	14	14	100	1.0	1.0	1.0
	**Personal Hygiene**	154	158	97.84	0.97	1.0	0.99
	**Cook Lunch**	64	64	100	1.0	1.0	1.0
HH125	**Eat Lunch**	7	1	97.93	1.0	0.14	0.25
	**Personal Hygiene**	219	225	97.93	0.97	1.0	0.99

## References

[1] Bandodkar A.J., Wang J. (2014). Non-invasive wearable electrochemical sensors: A review. Trends Biotechnol..

[2] Ioan S., Luminita D., Mihai T., Emilia P., Dan M. (2019). Unobtrusive Monitoring the Daily Activity Routine of Elderly People Living Alone, with Low-Cost Binary Sensors. Sensors.

[3] Krishnan N.C., Cook D.J. (2014). Activity recognition on streaming sensor data. Pervasive Mob. Comput..

[4] Benndorf M., Ringsleben F., Haenselmann T., Yadav B., Eibl M., Gaedke M. (2017). Automated Annotation of Sensor data for Activity Recognition using Deep Learning. INFORMATIK 2017.

[5] Chen B., Fan Z., Cao F. Activity Recognition Based on Streaming Sensor Data for Assisted Living in Smart Homes. Proceedings of the 2015 International Conference on Intelligent Environments.

[6] Yan S., Liao Y., Feng X., Liu Y. Real time activity recognition on streaming sensor data for smart environments. Proceedings of the 2016 International Conference on Progress in Informatics and Computing (PIC).

[7] Tapia E.M., Intille S.S., Larson K., Ferscha A., Mattern F. (2004). Activity Recognition in the Home Using Simple and Ubiquitous Sensors. Pervasive Computing.

[8] Kashimoto Y., Hata K., Suwa H., Fujimoto M., Arakawa Y., Shigezumi T., Komiya K., Konishi K., Yasumoto K. Low-cost and Device-free Activity Recognition System with Energy Harvesting PIR and Door Sensors. Proceedings of the 13th Annual International Conference on Mobile and Ubiquitous Systems: Computing, Networking and Services.

[9] Lu H., Yang J., Liu Z., Lane N.D., Choudhury T., Campbell A.T. The Jigsaw Continuous Sensing Engine for Mobile Phone Applications. Proceedings of the 8th ACM Conference on Embedded Networked Sensor Systems, 2010, SenSys’10.

[10] Stikic M., Larlus D., Ebert S., Schiele B. (2011). Weakly Supervised Recognition of Daily Life Activities with Wearable Sensors. IEEE Trans. Pattern Anal. Mach. Intell..

[11] Miluzzo E., Cornelius C.T., Ramaswamy A., Choudhury T., Liu Z., Campbell A.T. Darwin Phones: The Evolution of Sensing and Inference on Mobile Phones. Proceedings of the 8th International Conference on Mobile Systems, Applications, and Services, 2010, MobiSys ’10.

[12] U.S. BUREAU OF LABOR STATISTICS (2018). American Time Use Survey Activity Lexicon.

[13] Alabdulmohsin I.M., Cissé M., Zhang X. Is Attribute-Based Zero-Shot Learning an Ill-Posed Strategy?. Proceedings of the ECML-PKDD 2016: European Conference on Machine Learning and Principles and Practice of Knowledge Discovery.

[14] Fu Y., Hospedales T.M., Xiang T., Gong S. (2015). Transductive Multi-view Zero-Shot Learning. arXiv.

[15] Wang W., Zheng V.W., Yu H., Miao C. (2019). A Survey of Zero-Shot Learning: Settings, Methods, and Applications. ACM TIST.

[16] Mikolov T., Sutskever I., Chen K., Corrado G., Dean J. (2013). Distributed Representations of Words and Phrases and their Compositionality. arXiv.

[17] Mikolov T., Chen K., Corrado G.S., Dean J. (2013). Efficient Estimation of Word Representations in Vector Space. arXiv.

[18] De Souza Júnior A.H., Corona F., Barreto G.D.A., Miché Y., Lendasse A. (2015). Minimal Learning Machine: A novel supervised distance-based approach for regression and classification. Neurocomputing.

[19] Botros M. (2017). Supervised Learning in Human Activity Recognition Based on Multimodal Body Sensing. Bachelor’s Thesis.

[20] Nabian M. (2017). A Comparative Study on Machine Learning Classification Models for Activity Recognition. J. Inf. Technol. Softw. Eng..

[21] He J., Zhang Q., Wang L., Pei L. (2019). Weakly Supervised Human Activity Recognition from Wearable Sensors by Recurrent Attention Learning. IEEE Sens. J..

[22] Kharat M.V., Walse K.H., Dharaskar D.R.V. (2017). Survey on Soft Computing Approaches for Human Activity Recognition. Int. J. Sci. Res..

[23] Qian H., Pan S.J., Miao C. (2019). Distribution-Based Semi-Supervised Learning for Activity Recognition.

[24] Zhu Q., Chen Z., Soh Y.C. (2019). A Novel Semisupervised Deep Learning Method for Human Activity Recognition. IEEE Trans. Ind. Informat..

[25] Chen W.H., Cho P.C., Jiang Y.L. (2017). Activity Recognition Using Transfer Learning. Sens. Mater..

[26] Cook D.J., Feuz K.D., Krishnan N.C. (2013). Transfer learning for activity recognition: A survey. Knowl. Inf. Syst..

[27] Hu D. Transfer learning for activity recognition via sensor mapping. Proceedings of the Twenty-Second International Joint Conference on Artificial Intelligence.

[28] Bulling A., Blanke U., Schiele B. (2014). A tutorial on human activity recognition using body-worn inertial sensors. ACM Comput. Surv..

[29] Hu N., Lou Z., Englebienne G., Kròse B.J.A. (2014). Learning to Recognize Human Activities from Soft Labeled Data. Robot. Sci. Syst..

[30] Alex P.M.D., Ravikumar A., Selvaraj J., Sahayadhas A. (2018). Research on Human Activity Identification Based on Image Processing and Artificial Intelligence. Int. J. Eng. Technol..

[31] Jaouedi N., Boujnah N., Bouhlel M.S. (2019). A new hybrid deep learning model for human action recognition. J. King Saud Univ. Comput. Inf. Sci..

[32] Antón M.Á., Meré J.B.O., Saralegui U., Sun S. (2019). Non-Invasive Ambient Intelligence in Real Life: Dealing with Noisy Patterns to Help Older People. Sensors.

[33] Shahmohammadi F., Hosseini A., King C.E., Sarrafzadeh M. Smartwatch Based Activity Recognition Using Active Learning. Proceedings of the 2017 IEEE/ACM International Conference on Connected Health: Applications, Systems and Engineering Technologies (CHASE).

[34] Bulbul E., Cetin A., Dogru I.A. Human Activity Recognition Using Smartphones. Proceedings of the 2018 2nd International Symposium on Multidisciplinary Studies and Innovative Technologies (ISMSIT).

[35] Laput G., Zhang Y., Harrison C. Synthetic Sensors: Towards General-Purpose Sensing. Proceedings of the 2017 CHI Conference on Human Factors in Computing Systems, CHI ’17.

[36] Chung S., Lim J., Noh K.J., Kim G., Jeong H. (2019). Sensor Data Acquisition and Multimodal Sensor Fusion for Human Activity Recognition Using Deep Learning. Sensors.

[37] Balli S., Sağbaş E.A., Peker M. (2018). Human activity recognition from smart watch sensor data using a hybrid of principal component analysis and random forest algorithm. Meas. Control..

[38] Hsu Y.L., Yang S.C., Chang H.C., Lai H.C. (2018). Human Daily and Sport Activity Recognition Using a Wearable Inertial Sensor Network. IEEE Access.

[39] Ilbeygi M., Kangavari M.R. (2018). Comprehensive architecture for intelligent adaptive interface in the field of single-human multiple-robot interaction. ETRI J..

[40] Dharmalingam S., Palanisamy A. (2018). Vector space based augmented structural kinematic feature descriptor for human activity recognition in videos. ETRI J..

[41] Moon J., Jin J., Kwon Y., Kang K., Park J., Park K. (2017). Extensible Hierarchical Method of Detecting Interactive Actions for Video Understanding. ETRI J..

[42] Zheng Y., Ding X.R., Poon C.C.Y., Lo B.P.L., Zhang H., Zhou X.L., Yang G.Z., Zhao N., Zhang Y.T. (2014). Unobtrusive Sensing and Wearable Devices for Health Informatics. IEEE Trans. Biomed. Eng..

[43] Jalal A., Kim Y., Kim Y.J., Kamal S., Kim D. (2017). Robust human activity recognition from depth video using spatiotemporal multi-fused features. Pattern Recognit..

[44] Stankovic J.A., Srinivasan V. (2012). Non-Invasive Sensor Solutions for Activity Recognition in Smart Homes.

[45] Bhandari B., Lu J., Zheng X., Rajasegarar S., Karmakar C.K. Non-invasive sensor based automated smoking activity detection. Proceedings of the 2017 39th Annual International Conference of the IEEE Engineering in Medicine and Biology Society (EMBC).

[46] Štulienė A., Paulauskaite-Taraseviciene A. (2017). Research on human activity recognition based on image classification methods. Comput. Sci..

[47] Alsheikh M.A., Selim A., Niyato D., Doyle L., Lin S., Tan H.P. (2015). Deep Activity Recognition Models with Triaxial Accelerometers. arXiv.

[48] Ronao C.A., Cho S.B. (2016). Human activity recognition with smartphone sensors using deep learning neural networks. Expert Syst. Appl..

[49] Bhattacharya S., Lane N.D. From smart to deep: Robust activity recognition on smartwatches using deep learning. Proceedings of the 2016 IEEE International Conference on Pervasive Computing and Communication Workshops (PerCom Workshops).

[50] Zhang L., Xiang T., Gong S. Learning a Deep Embedding Model for Zero-Shot Learning. Proceedings of the 2017 IEEE Conference on Computer Vision and Pattern Recognition (CVPR).

[51] Larochelle H., Erhan D., Bengio Y. (2008). Zero-Data Learning of New Tasks.

[52] Lampert C.H., Nickisch H., Harmeling S. Learning to detect unseen object classes by between-class attribute transfer. Proceedings of the 2009 IEEE Conference on Computer Vision and Pattern Recognition.

[53] Palatucci M., Pomerleau D., Hinton G.E., Mitchell T.M. Zero-Shot Learning with Semantic Output Codes. Proceedings of the Neural Information Processing Systems Conference, NIPS.

[54] Cheng H.T., Sun F.T., Griss M.L., Davis P., Li J., You D. NuActiv: Recognizing unseen new activities using semantic attribute-based learning. Proceedings of the 11th Annual International Conference on Mobile Systems, Applications, and Services, MobiSys.

[55] Cheng H.T., Griss M.L., Davis P., Li J., You D. Towards zero-shot learning for human activity recognition using semantic attribute sequence model. Proceedings of the 2013 ACM International Joint Conference on Pervasive and Ubiquitous Computing, UbiComp.

[56] Wijekoon A., Wiratunga N., Sani S. Zero-Shot Learning with Matching Networks for Open-Ended Human Activity Recognition. Proceedings of the 2013 ACM International Joint Conference on Pervasive and Ubiquitous Computing, SICSA ReaLX 2018.

[57] Roitberg A., Martinez M., Haurilet M., Stiefelhagen R. Towards a Fair Evaluation of Zero-Shot Action Recognition Using External Data. Proceedings of the ECCV 2018: European Conference on Computer Vision.

[58] Machot F., Mayr H.C., Michael J., Ali M., Pan J.S., Chen S.M., Horng M.F. (2014). Behavior Modeling and Reasoning for Ambient Support: HCM-L Modeler. Modern Advances in Applied Intelligence.

[59] Zellers R., Choi Y. Zero-Shot Activity Recognition with Verb Attribute Induction. Proceedings of the EMNLP 2017: Empirical Methods in Natural Language Processing.

[60] Gao J., Zhang T., Xu C. I Know the Relationships: Zero-Shot Action Recognition via Two-Stream Graph Convolutional Networks and Knowledge Graphs. Proceedings of the AAAI.

[61] Mishra A., Verma V.K., Reddy M.S.K., Subramaniam A., Rai P., Mittal A. A Generative Approach to Zero-Shot and Few-Shot Action Recognition. Proceedings of the 2018 IEEE Winter Conference on Applications of Computer Vision (WACV).

[62] Google-News-Embedding (2013). Google Code Archive—Long-Term Storage for Google Code. https://code.google.com/archive/p/word2vec/.

[63] Hornik K., Stinchcombe M., White H. (1989). Multilayer feedforward networks are universal approximators. Neural Netw..

[64] Funahashi K.I. (1989). On the approximate realization of continuous mappings by neural networks. Neural Netw..

[65] Kingma D.P., Ba J. (2014). Adam: A method for stochastic optimization. arXiv.

[66] Powers D.M. (2011). Evaluation: From precision, recall and F-measure to ROC, informedness, markedness and correlation. J. Mach. Learn. Technol..

[67] Dinu G., Baroni M. (2014). Improving zero-shot learning by mitigating the hubness problem. arXiv.

[68] Radovanovic M., Nanopoulos A., Ivanovic M. (2010). Hubs in Space: Popular Nearest Neighbors in High-Dimensional Data. J. Mach. Learn. Res..

[69] Shigeto Y., Suzuki I., Hara K., Shimbo M., Matsumoto Y. Ridge Regression, Hubness, and Zero-Shot Learning. Proceedings of the sof European Conference on Machine Learning and Principles and Practice of Knowledge Discovery in Databases (ECML PKDD).

[70] Paul A., Krishnan N.C., Munjal P. Semantically Aligned Bias Reducing Zero Shot Learning. Proceedings of the CVPR 2019.

[71] Song J., Shen C., Yang Y., Liu Y.P., Song M. Transductive Unbiased Embedding for Zero-Shot Learning. Proceedings of the 2018 IEEE/CVF Conference on Computer Vision and Pattern Recognition.

[72] Machot F.A., Mosa A.H., Ali M., Kyamakya K. (2018). Activity Recognition in Sensor Data Streams for Active and Assisted Living Environments. IEEE Trans. Circuits Syst. Video Technol..

[73] Du Y., Lim Y., Tan Y. (2019). A Novel Human Activity Recognition and Prediction in Smart Home Based on Interaction. Sensors.

[74] Cao X.H., Obradovic Z., Kim K. A Simple yet Effective Model for Zero-Shot Learning. Proceedings of the 2018 IEEE Winter Conference on Applications of Computer Vision (WACV).

[75] Krizhevsky A. (2009). Learning Multiple Layers of Features from Tiny Images. Master’s Thesis.

[76] Farhadi A., Endres I., Hoiem D., Forsyth D. Describing objects by their attributes. Proceedings of the 2009 IEEE Conference on Computer Vision and Pattern Recognition.

[77] Wah C., Branson S., Welinder P., Perona P., Belongie S. (2011). The Caltech-UCSD Birds-200–2011 Dataset.

[78] Patterson G., Xu C., Su H., Hays J. (2014). The sun attribute database: Beyond categories for deeper scene understanding. Int. J. Comput. Vis..

